# Epigallocatechin-3-gallate Protects against Hydrogen Peroxide-Induced Inhibition of Osteogenic Differentiation of Human Bone Marrow-Derived Mesenchymal Stem Cells

**DOI:** 10.1155/2016/7532798

**Published:** 2016-02-09

**Authors:** Dawei Wang, Yonghui Wang, Shihong Xu, Fu Wang, Bomin Wang, Ke Han, Daqing Sun, Lianxin Li

**Affiliations:** ^1^Department of Orthopedics, Shandong Provincial Hospital Affiliated to Shandong University, Jinan 250021, China; ^2^Department of Vascular Surgery, Second Hospital of Shandong University, Jinan 250000, China

## Abstract

Oxidative stress induces bone loss and osteoporosis, and epigallocatechin-3-gallate (EGCG) may be used to combat these diseases due to its antioxidative property. Herein, oxidative stress in human bone marrow-derived mesenchymal stem cells (BM-MSCs) was induced by H_2_O_2_, resulting in an adverse effect on their osteogenic differentiation. However, this H_2_O_2_-induced adverse effect was nullified when the cells were treated with EGCG. In addition, treatment of BM-MSCs with EGCG alone also resulted in the enhancement of osteogenic differentiation of BM-MSCs. After EGCG treatment, expressions of *β*-catenin and cyclin D1 were upregulated, suggesting that the Wnt pathway was involved in the effects of EGCG on the osteogenic differentiation of BM-MSCs. This was also confirmed by the fact that the Wnt pathway inhibitor, Dickkopf-1 (DKK-1), can nullify the EGCG-induced enhancement effect on BM-MSC's osteogenic differentiation. Hence, our results suggested that EGCG can reduce the effects of oxidative stress on Wnt pathway in osteogenic cells, which supported a potentially promising therapy of bone disorders induced by oxidative stress. Considering its positive effects on BM-MSCs, EGCG may also be beneficial for stem cell-based bone repair.

## 1. Introduction

Several types of polyphenols, including green tea polyphenols, grape polyphenols, and blueberry polyphenols, have been reported to be capable of promoting bone formation, preventing bone loss, and influencing osteogenic differentiation [1–4]. Epigallocatechin-3-gallate (EGCG) is the most abundant catechin polyphenol extracted from green tea, constituting 9–13% of the total dry weight [5]. After oral administration, EGCG is predominantly absorbed in the small intestine followed by transportation to other organs. Thus, due to its antioxidant and free radical scavenger properties, EGCG has been proposed to have protective effects for organ impairments induced by ischemia, toxins, stress, and hypertension [6, 7].

Oxidative stress may be associated with the pathophysiology of many organs. In bone tissues, generation of reactive oxygen species (ROS) can influence the homeostasis, since ROS contributes to bone remodeling by promoting bone resorption [8]. Recent studies suggested that some bone pathogenesis, such as osteoporosis, diabetes-induced bone complications, bone tumor development, and joint inflammatory diseases, may be associated with oxidative stress [9].

EGCG is beneficial for bone regeneration, possibly due to its ability to suppress bone resorption and inhibit functions of osteoclasts. EGCG has been shown to induce apoptotic cell death in cultured mouse osteoclasts and inhibit the formation of osteoclasts in a coculture system of mouse bone marrow cells and calvarial primary osteoblasts [10]. In addition, it has also been reported that EGCG can increase the differentiation of human osteoblast-like cells by reducing the expression of Runt-related transcription factor 2 (*Runx2*) in the late growth stage [11].

Bone marrow-derived mesenchymal stem cells (BM-MSCs) are an attractive cell source for bone defect repair, due to their relatively simple isolation and expansion procedures, as well as their pluripotent differentiation ability into mesenchymal tissues [12]. BM-MSCs are commonly used* ex vivo* in combination with three-dimensional (3D) porous biomaterial carriers to form a bone construct, which can be implanted to the defect sites to facilitate repair process by subsequent osteogenesis of the construct [13–15]. In addition, BM-MSCs can also be used in the absence of biomaterial carriers. Direct infusion of BM-MSCs systemically or through intrabone has been used to treat various degenerative bone disorders, such as osteogenesis imperfecta and osteoporosis [16–18].

This study aimed to investigate the potential protective roles of EGCG against the adverse effects of oxidative stress on osteogenic differentiation of BM-MSCs, as well as the possible underlying mechanisms. Oxidative stress in BM-MSCs was induced by H_2_O_2_, and the combined treatment of BM-MSCs with EGCG and H_2_O_2_ was used to investigate the potential effects of EGCG against oxidative stress. The possible involvement of Wnt pathway in the antioxidative effects of EGCG was also investigated.

## 2. Materials and Methods

### 2.1. Isolation and Culture of Human BM-MSC

Human BM-MSCs were isolated from marrow extracts harvested from a healthy donor without specific metabolic or inherited diseases. The marrow extracts were obtained with informed consent via routine iliac bone graft procedure for reconstruction of bone defects resulting from traumatic tibial fracture. The bone marrow tissue was washed out with growth medium containing Alpha-Modified Eagle's Medium (*α*-MEM; Gibco, USA) supplemented with 10% fetal bovine serum (FBS; Hyclone, USA), 100 U/L penicillin, and 100 U/L streptomycin (Solarbio, China). After centrifugation, the cells were suspended in the growth medium and maintained under a humidified atmosphere with 5% CO_2_ at 37°C. After 24 h of culture, the medium was refreshed to remove nonadherent cells. BM-MSCs were used when the culture reached 80–90% confluence. This study was approved by the ethics committee of Shandong Provincial Hospital affiliated to Shandong University.

### 2.2. Flow Cytometry

The expression of surface markers CD29, CD44, and CD105 (as positive markers) and CD34 (as a negative marker) on the isolated BM-MSCs was tested using the following antibodies (all purchased from Abcam, UK): FITC-conjugated mouse anti-human CD29, FITC-conjugated mouse monoclonal anti-human CD44, FITC-conjugated mouse monoclonal anti-human CD105, and FITC-conjugated mouse monoclonal anti-human CD34. Flow cytometric analysis was conducted on a FACSCalibur platform (BD Biosciences, NJ). Unstained cells were used for gate setting, and at least 15,000 events were collected for analysis of each sample.

### 2.3. Treatment of BM-MSCs with H_2_O_2_, EGCG, and Dickkopf-1 (DKK-1)

To induce osteogenic differentiation of BM-MSCs, the cells were cultured in differentiation medium containing *α*-MEM medium supplemented with 10% FBS, 50 *μ*g/mL L-ascorbic acid (Sigma, USA), 10 mM *β*-glycerophosphate (Sigma), and 100 nM dexamethasone (Sigma). Various amounts of H_2_O_2_ and/or EGCG (Sigma) were added to the differentiation medium for investigation of their effects on M-MSCs. Cells cultured in medium without H_2_O_2_ and EGCG were used as control. To investigate whether Wnt pathway is involved in osteogenic differentiation of BM-MSCs, 0.2 *μ*g/mL Wnt inhibitor, Dickkopf-1 (DKK-1; Peprotech, USA), was added to the culture medium containing 0.2 mM H_2_O_2_ and 5 *μ*M EGCG.

### 2.4. Cell Viability Assay

Cell viability was measured using Cell Counting Assay Kit 8 (CCK-8; Sigma, USA) according to the manufacturer's protocol. BM-MSCs were seeded onto 96-well plates (5 × 10^3^ cells/well) and maintained in growth medium overnight. After removal of the medium, fresh medium containing different concentrations of H_2_O_2_ and/or EGCG was added. After incubation for 24 h, 10 *μ*L of CCK-8 solution was added to each well to incubate for 1 h, and the absorbance at 450 nm was measured with a microplate reader (Molecular Devices, USA).

### 2.5. Glutathione (GSH) Level Measurement

To measure GSH level, the cells were lysed with 200 *μ*L of RIPA Lysis Buffer (Beyotime Institute of Biotechnology, China) containing 1 mM phenylmethanesulfonyl fluoride (PMSF). 20 *μ*L of cell lysate was treated with 5% trichloroacetic acid and then mixed with 660 *μ*L of 67 mM sodium and potassium phosphate buffer (pH = 8) and 330 *μ*L of 1 mM 5,5′-dithiobis(2-nitrobenzoate) (DTNB). The samples were incubated at room temperature in the dark for 45 min and the absorbance at 412 nm was measured. GSH concentration was calculated based on a calibration curve obtained using commercial standards.

### 2.6. Superoxide Dismutase (SOD) Activity and Malondialdehyde (MDA) Level Measurement

SOD activity was measured using a Superoxide Dismutase (ransod) Diluent Assay kit (Randox Laboratories Ltd.) according to the manufacturer's instruction. MDA level was measured with detection kits (Jiancheng Biotech, Nanjing, China) according to the manufacturer's instructions. Briefly, 100 mL thiobarbituric acid (TBA) solution containing 15 mL of 100% trichloroacetic acid, 0.375 g of TBA, 25 mL of 1 N hydrochloric acid (HCl), and 40 mg of butylated hydroxytoluene (BHT)/ethanol was freshly prepared. 1 mL of TBA solution and 15 *μ*L of 50 mM BHT were added to 0.5 mL of samples. The mixture was heated at 95°C for 45 min and then cooled in ice-cold water to measure the absorbance at 532 nm on a spectrophotometer.

### 2.7. Alkaline Phosphatase (ALP) Activity Assay

To examine the effect of EGCG on the ALP activity of BM-MSCs, 3 × 10^4^ cells were seeded on 24-well microplate and cultured in the osteogenic differentiation medium (as mentioned above) containing various concentrations of EGCG. After 8 days of culture, cells were washed with phosphate-buffered saline (PBS) and stained with an ALP staining kit (Nanjing Jiancheng Bioengineering Research Institute, China) following the manufacturer's instructions. The staining was then observed under a microscope (IX71, Olympus, Japan). To quantify the ALP activity, the cells were lysed with 200 *μ*L of RIPA Lysis Buffer (Beyotime Institute of Biotechnology, China) containing 1 mM phenylmethanesulfonyl fluoride (PMSF), followed by measuring the ALP activity (units/mL) with an ALP reagent kit (Nanjing Jiancheng Bioengineering Research Institute) as per the manufacturer's instructions.

### 2.8. Calcium Accumulation Assay

Calcium accumulation assay was conducted using Alizarin Red staining. After 16 days of culture, cells were washed with distilled water and fixed with ice-cold 70% (v/v) ethanol for 1 h. After rising twice with deionized water, the cells were stained with Alizarin Red S (ARS; Sigma, USA) solution (40 mM in TRIS buffer, pH 4.2) for 10 min at room temperature, and the excess dye was gently washed away with water. The stained calcification deposits (red) were examined under a microscope (Olympus, Japan). The Alizarin Red-sulfate staining density was determined by melting the stained sample using 10% (w/v) cetylpyridinium chloride dissolved in 10 mM sodium phosphate (pH 7.0) and measuring at 562 nm.

### 2.9. Quantitative Reverse Transcription Polymerase Chain Reaction (qRT-PCR)

qRT-PCR was performed for the detection of the osteogenic gene expression of cells cultured in 6-well plates. Total RNA was harvested with a Reverse Transcription System (Promega, USA) according to the manufacturer's instructions, and 1 mg of the extracted total RNA was used for cDNA synthesis. qRT-PCR was conducted on an ABI 7300 Sequence Detection System (Applied Biosystems, USA) using TaqMan Universal PCR Master Mix (Applied Biosystems, USA). The primers used are* Runx2* (forward: 5′-TGTCATGGCGGGTAACGATG-3′, reverse: 5′-CCCTAAATCACTGAGGCGGT-3′),* osterix* (*OSX*, forward: 5′-CCTCTGCGGGACTCAACAAC-3′, reverse: 5′-AGCCCATTAGTGCTTGTAAAGG-3′), *β-catenin* (forward: 5′-AAAGCGGCTGTTAGTCACTGG-3′, reverse: 5′-CGAGTCATTGCATACTGTCCAT-3′),* cyclin D1* (forward: 5′-GCTGCGAAGTGGAAACCATC-3′, reverse: 5′-CCTCCTTCTGCACACATTTGAA-3′), and* glyceraldehyde-3-phosphate dehydrogenase* (*GAPDH*, forward: 5′-CTATAAATTGAGCCCGCAGC-3′, reverse: 5′-GACCAAATCCGTTGACTCCG-3′). Gene expression level was calculated using 2^−ΔΔCt^ method with* GAPDH* as the reference gene.

### 2.10. Western Blotting

Homogenization of BM-MSCs was conducted with a Polytron homogenizer for 10 s in cold Pro-PREP protein extraction solution (Intron, Seongnam, Korea) containing 1 mM pepstatin A, 1 mM PMSF, 0.1 mM aprotinin, 1 mM leupeptin, and 1 mM EDTA. Cell lysates were kept at −20°C for 30 min. After centrifugation for 10 min at 10,000 ×g, the concentration of proteins in the supernatant was determined with an enhanced BCA protein assay kit (Beyotime Institute of Biotechnology). After boiling in 5x sample buffer (50 mM Tris, 2% (w/v) SDS, 5% (v/v) glycerol, and 10% (v/v) 2-mercaptoethanol, pH 6.8), 50 mg of the total proteins was separated by molecular mass on 12% (w/v) sodium dodecyl sulfate-polyacrylamide gel electrophoresis (SDS-PAGE) gels, with 5% (w/v) polyacrylamide stacking gels. The expression levels of the following proteins were determined: *β*-catenin (88 kDa), cyclin D1 (35 kDa), and GAPDH (37 kDa).

Separated proteins were transferred onto polyvinylidene fluoride (PVDF) membranes (Bio-Rad), which were then blocked with 5% (w/v) skim milk in Tris-buffered saline containing 0.1% (v/v) Tween 20, and incubated with commercial primary antibodies (1 : 1,000 dilution; all purchased from Abcam, UK): mouse monoclonal anti-human GAPDH, rabbit monoclonal anti-human *β*-catenin, and rabbit monoclonal anti-human cyclin D1. Blots were then incubated with secondary horseradish peroxidase-conjugated anti-mouse or anti-rabbit antibodies (1 : 5,000 dilutions; Abcam) followed by development with an enhanced chemiluminescence detection kit (Millipore, USA). Signal intensity was quantified by an EZ-Capture II chemiluminescence imaging system with a charge-cooled camera (Atto Corp., Japan). Relative protein levels were expressed as the ratio of the intensity of each protein to the intensity of GAPDH.

### 2.11. Statistical Analysis

For each condition and time point, a minimum of three independent experiments were carried out. One- or two-way analysis of variance (ANOVA) with Tukey* post hoc* test was used to evaluate the data and the results are presented as mean ± SEM. Statistical significance was accepted at *p* < 0.05.

## 3. Results

### 3.1. Effects of H_2_O_2_ and EGCG on BM-MSC's Viability

**Figure 1:**
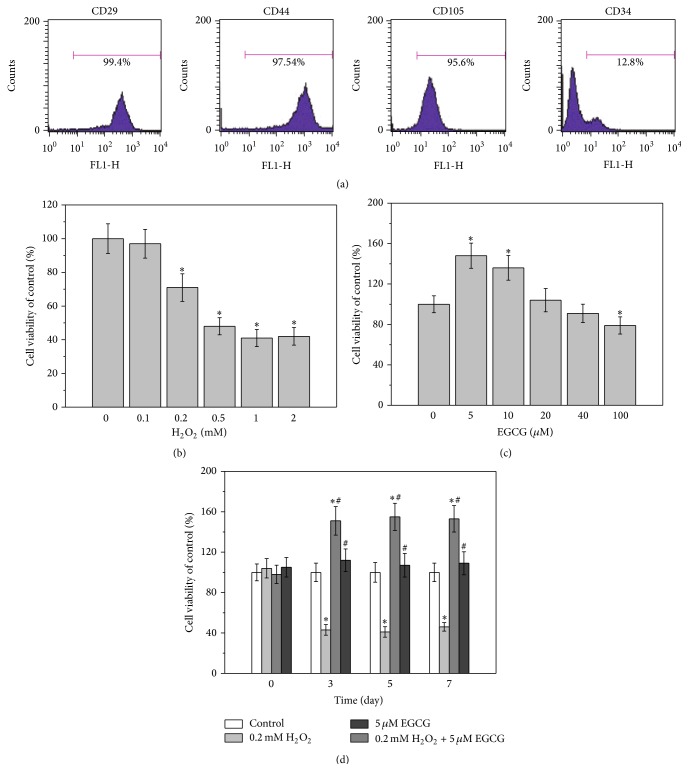
Effects of H_2_O_2_ and EGCG on cell viability of human BM-MSCs. (a) Characterization of human BM-MSCs by flow cytometry. The majority of the cells are CD29+, CD44+, CD105+, and CD34−, which are typical characteristic phenotypes of BM-MSCs. Effects of different concentrations of H_2_O_2_ exposure (0.1 to 2 mM) (b) or EGCG exposure (5 to 100 *μ*M) (c) for 24 h on cell viability of BM-MSCs, measured by MTT assay. (d) Cotreatment of 0.2 mM H_2_O_2_ and 5 *μ*M EGCG for indicated time on cell viability of BM-MSCs. Data were presented as mean ± SEM. ^*∗*^ *p* < 0.05 versus control and ^#^ *p* < 0.01 versus 0.2 mM H_2_O_2_ treatment alone.

The isolated human BM-MSCs were identified by flow cytometry analysis for expressions of common surface markers of MSCs. As shown in Figure 1(a), the majority of the cells expressed CD29, CD44, and CD105, whereas few cells expressed CD34. This indicated that the isolated cells were indeed BM-MSCs. CCK-8 assay results demonstrated that the viability of BM-MSCs was reduced with increasing concentrations of H_2_O_2_ in the cell culture medium. The viability of BM-MSCs started to decrease when treated with 0.2 mM H_2_O_2_, and the IC_50_ value was approximately 0.5 mM (Figure 1(b)). The concentration-dependent effects of EGCG on the viability of BM-MSCs were also investigated. Incubation with 5 and 10 *μ*M EGCG for 24 h significantly enhanced cell viability, whereas further elevation in EGCG concentration did not affect the viability of the cells (Figure 1(c)). Therefore, 5 *μ*M EGCG was selected as the optimal concentration for subsequent experiments. BM-MSCs were then treated with the combination of 0.2 mM H_2_O_2_ and 5 *μ*M EGCG, and the results showed that EGCG almost completely nullified the H_2_O_2_-induced reduction on cell viability over 7 days (Figure 1(d)).

### 3.2. Effects of H_2_O_2_ and EGCG on the Oxidative Stress Status of BM-MSCs

**Figure 2:**
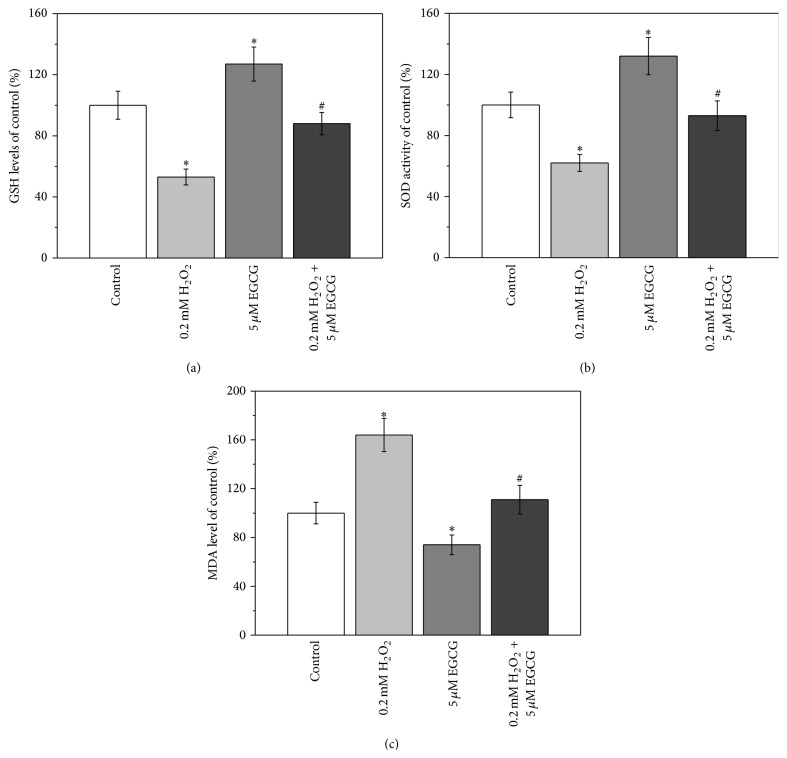
5 *μ*M EGCG attenuates 0.2 mM H_2_O_2_-induced oxidative stress in human BM-MSCs. GSH levels (a), SOD activity (b), and MDA levels (c) were shown in the cells when cultured for 7 days. Data were presented as mean ± SEM. ^*∗*^ *p* < 0.05 versus control and ^#^ *p* < 0.05 versus 0.2 mM H_2_O_2_ treatment alone.

**Figure 3:**
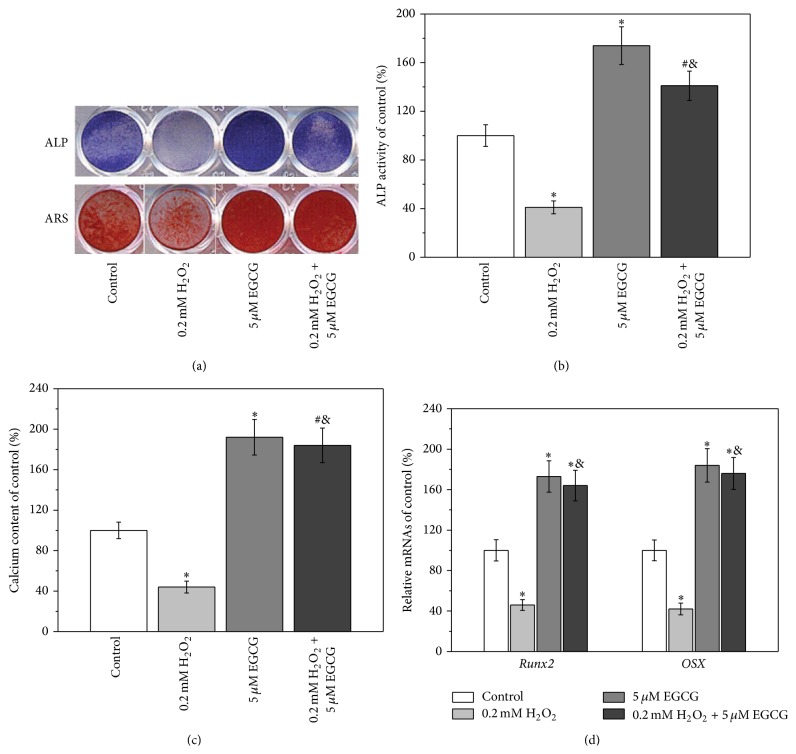
5 *μ*M EGCG protects against 0.2 mM H_2_O_2_-induced inhibition of osteogenic differentiation of human BM-MSCs. (a) Representative ALP and ARS staining images in the four experimental groups. ALP activity (b) and calcium contents (c) of the cultures of the experimental groups normalized to control. (d) Relative mRNAs expression of* Runx2* and *OSX* in the experimental groups, quantified by RT-PCR. Data were presented as mean ± SEM. ^*∗*^ *p* < 0.01 and ^#^ *p* < 0.05 versus control, and ^&^ *p* < 0.01 versus 0.2 mM H_2_O_2_ treatment alone.

As shown in Figure 2(a), GSH level was reduced when BM-MSCs were treated with 0.2 mM H_2_O_2_, whereas treatment with 5 *μ*M EGCG significantly elevated the GSH level. When the cells were cotreated with H_2_O_2_ and EGCG, the GSH level was restored to the same level as the control group. Similar trends in the changes of SOD levels were also observed, when BM-MSCs were treated with H_2_O_2_, EGCG alone, and combined treatment with H_2_O_2_ and EGCG, respectively (Figure 2(b)). However, the situation was different for MDA level. Treatment of BM-MSCs with 0.2 mM H_2_O_2_ resulted in an increase in the MDA level, whereas when the cells were treated with 5 *μ*M EGCG, the MDA level was lower than that of control (Figure 3(c)). The combinational treatment with EGCG and H_2_O_2_ restored MDA level similar to that of control.

### 3.3. Effects of H_2_O_2_ and EGCG on the Osteogenic Differentiation of BM-MSCs

As compared with control, when BM-MSCs were treated with 0.2 mM H_2_O_2_, the extent of ALP and ARS staining was significantly reduced, whereas treatment with EGCG resulted in enhanced staining (Figure 3(a)). When BM-MSCs were cotreated with H_2_O_2_ and EGCG, the extent of ALP and ARS staining became significantly higher than that of control (Figure 3(a)). Quantitative analysis of ALP activity and calcium deposition showed similar results (Figures 3(b) and 3(c)). As compared with control, treatment with 0.2 mM H_2_O_2_ resulted in approximately 60% decrease in the ALP activity and calcium deposition content. When the cells were cotreated with H_2_O_2_ and EGCG, the ALP activity increased by approximately 30%, and calcium deposition content increased by approximately 70%. The expression levels of osteogenic markers of BM-MSCs after the different treatment were also investigated, and similar results were obtained (Figure 3(d)).

### 3.4. Involvement of Wnt Pathway in the Protective Effects of EGCG from Oxidative Stress

**Figure 4:**
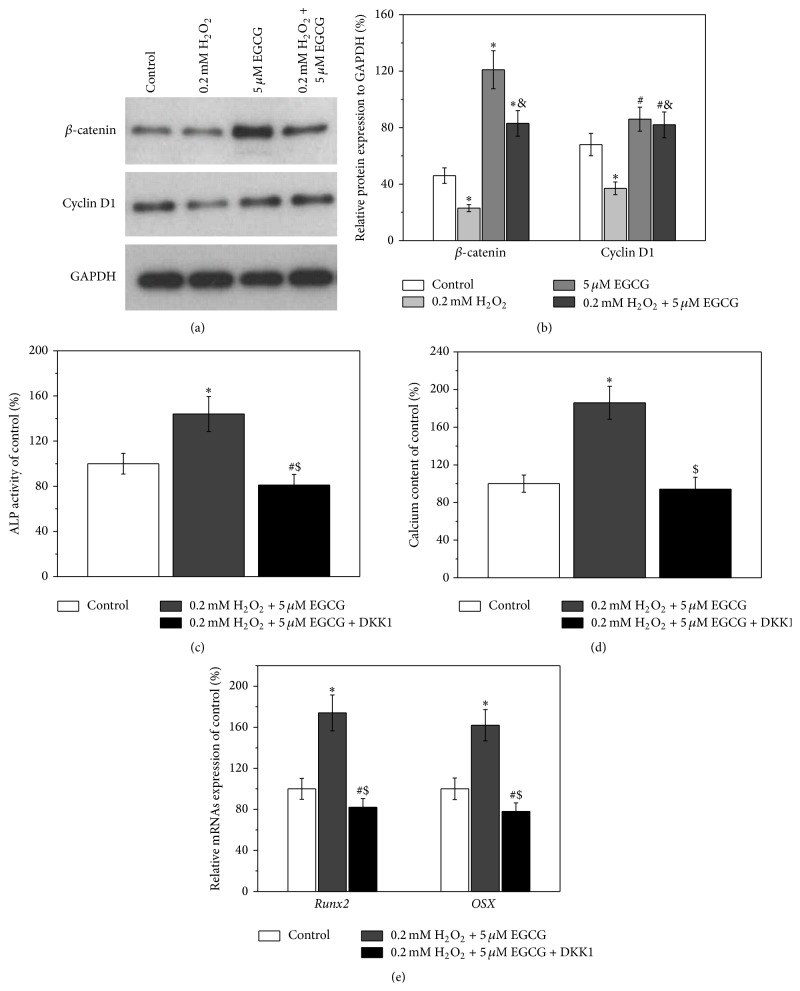
5 *μ*M EGCG rescues the inhibition of osteogenic differentiation induced by 0.2 mM H_2_O_2_ involved in the Wnt pathway. (a) Western blot analysis of Wnt pathway-related regulators *β*-catenin and cyclin D1 in the cultures of experimental groups. (b) Relative protein expressions normalized to GAPDH in (a). Wnt inhibitor DKK-1 blocked the protective effects of EGCG on the inhibition of osteogenic differentiation by 0.2 mM H_2_O_2_ exposure, as evidenced by ALP activity (c), calcium contents (d), and relative mRNAs expression of* Runx2* and* OSX* (e). Data were presented as mean ± SEM. ^*∗*^ *p* < 0.01 and ^#^ *p* < 0.05 versus control, ^&^ *p* < 0.01 versus 0.2 mM H_2_O_2_ treatment alone in (b), and ^$^ *p* < 0.01 versus cotreatment of 0.2 mM H_2_O_2_ and 0.1 *μ*M EGCG.

As shown in Figure 4(a), Western blot results indicated that treatment of BM-MSCs with 0.2 mM H_2_O_2_ reduced the expression levels of  *β-catenin* and* cyclin D1*, while treatment with 5 *μ*M EGCG can nullify this downregulated effect induced by H_2_O_2_ by restoring the expression levels of these two regulators of Wnt pathway. Similar results were obtained by qRT-PCR analysis (Figure 4(b)). As described above, when BM-MSCs were cotreated with H_2_O_2_ and EGCG, the osteogenic differentiation of BM-MSCs was enhanced as compared to control. However, the enhancement effects were significantly reduced when DKK1 was added. The ALP activity, calcium deposition, and the expression levels of* Runx2* and *OSX* in the DKK-1-treated group were similar or even lower than those of control (Figures 4(c)–4(e)).

## 4. Discussion

MSCs are an attractive cell model for biomedical research over the past decade. MSCs are defined by positive expressions of CD29, CD44, CD73, CD90, and CD105 and negative expressions of CD19, CD34, and CD45 [19]. Thus, the positive expressions of CD29, CD44, and CD105 together with negative expressions of CD34 (Figure 1(a)) in our current study confirmed the identity of isolated cells as BM-MSCs.

H_2_O_2_ is harmful for human cells because it induces oxidative stress [20, 21]. As demonstrated by the cell viability assays, the damage induced by H_2_O_2_ on BM-MSCs was dose dependent, with the reduction beginning at 0.2 mM (Figure 1(b)). The enhancement of cell viability after treatment with 5 and 10 *μ*M EGCG was observed, which is consistent with previous study [22]. Since our aim was to investigate the protective effects of EGCG against oxidative stress damage, BM-MSCs were then treated with 5 *μ*M EGCG and 0.2 mM H_2_O_2_. The cell viability results clearly indicated that, under this condition, the protective effects of EGCG can be obviously observed (Figure 1(d)), which represented a reliable model for the following investigations.

The oxidative status of BM-MSCs after different treatments was further investigated using measurements of the levels of GSH, SOD activity, and MDA. GSH is a tripeptide, which acts as an important antioxidant to prevent damage to important cellular components induced by ROS [23]. SOD is another important antioxidant enzyme in living cells, which is able to catalyze the dismutation or partitioning of the superoxide (O_2_^−^) radical into other less harmful compounds such as H_2_O_2_ [24]. MDA is the product of the degradation of polyunsaturated lipids induced by ROS [25]. Therefore, the observed decrease in the levels of GSH and SOD activity, together with the increase in the MDA level, after treatment with H_2_O_2_ indicated that 0.2 mM H_2_O_2_ can induce oxidative stress in BM-MSCs (Figure 2). When BM-MSCs were treated with EGCG alone, the oxidative stress can be relieved. Furthermore, when the cells were cotreated with H_2_O_2_ and EGCG, the oxidative stress induced by H_2_O_2_ can no longer be observed (Figure 2).

After confirmation of the H_2_O_2_-induced oxidative stress and the antioxidative effect of EGCG, the effects of oxidative stress and EGCG on the osteogenic differentiation of BM-MSCs were subsequently investigated. ALP activity and calcium deposition are commonly used as indicators for early and late stages of osteogenic differentiation [26, 27], and* Runx2* and* OSX* are key transcription factors for osteogenic differentiation [28]. The results shown in Figure 3 indicated that the oxidative stress induced by H_2_O_2_ exhibited a negative effect on the osteogenic differentiation of BM-MSCs, which is consistent with previous study [29]. The positive effect of EGCG on BM-MSC's osteogenic differentiation was possibly attributed to the antioxidative property of EGCG [22]. Thus, when the cells were treated with H_2_O_2_ and EGCG, EGCG can nullify the H_2_O_2_-induced adverse effects on the osteogenic differentiation of BM-MSCs (Figure 3).

Oxidative stress can cause irreversible changes in cellular proteins, resulting in permanent loss of cellular functions or even cell death. Wnt pathway has been reported to be involved in the damage caused by oxidative stress [30]. Hence, the involvement of Wnt pathway in the protective effects of EGCG from oxidative stress induced by H_2_O_2_ and the effect on BM-MSC's osteogenic differentiation was investigated. *β*-catenin and cyclin D1 are two important regulators in Wnt pathway. Activation of Wnt pathway increases nuclear and cytoplasmic levels of *β*-catenin, which initiates transcriptional activation of* cyclin D1* and subsequently regulates cell behavior [31]. The results shown in Figures 4(a) and 4(b) suggested that Wnt pathway was involved in the EGCG-induced enhancement of osteogenic differentiation of BM-MSCs, which was consistent with a previous study that reported the involvement of Wnt pathway in osteoblast differentiation and bone formation [32]. This notion was further confirmed by the results obtained with DKK-1, an effective Wnt pathway inhibitor, which also nullified the EGCG-induced enhancement of osteogenic differentiation of BM-MSCs.

In fact, the discrepancies of experimental conditions in the current literatures contribute to the differences of reported biological effects of EGCG, including various administration routes,* in vitro* or* in vivo* studies, different cell lines, and different duration of exposure time. For* in vitro* observations, indeed there are many studies where EGCG at the concentrations below 1 *μ*M can exert certain effects, while there are also many studies where EGCG concentrations should be higher than 1 *μ*M, even around micro molarity, to exhibit significant effects [33–38]. For* in vivo* studies, although the mean levels of EGCG achievable in human plasma were reported to not exceed 0.2 *μ*M [39], the samples in this study were administrated by a single oral dose of EGCG (2 mg/kg). In fact, we may enhance the EGCG concentrations* in vivo* to a comparable concentration to our study by many ways, such as intraperitoneal injection and increasing the administration times and doses.

## 5. Conclusion

Oxidative stress on BM-MSCs can be induced by 0.2 mM H_2_O_2_, resulting in adverse effects on the viability and osteogenic differentiation, which can then be nullified by EGCG, as indicated by the results of ALP activity, calcium deposition, and the gene expressions of* Runx2* and* OSX*. In addition, EGCG alone exhibited positive effects on the functions of BM-MSCs. We propose that EGCG exhibits these antioxidative effects via Wnt pathway. In summary, our results promoted the current understanding of the antioxidative effects of EGCG and indicated EGCG as a promising treatment strategy for bone repair therapies.
